# Multisystem Inflammatory Syndrome Due to COVID-19 Infection in Vaccinated Versus Unvaccinated Adolescents: A Case Report

**DOI:** 10.7759/cureus.45564

**Published:** 2023-09-19

**Authors:** Miana R Zapata, Trupti Pandit, Lokesh Goyal, Kunal Ajmera, Prabal Chourasia, Ramesh Pandit

**Affiliations:** 1 Family Medicine, University of the Incarnate Word School of Osteopathic Medicine, San Antonio, USA; 2 Pediatrics, Nemours Children's Health, Glen Mills, USA; 3 Hospital Medicine, CHRISTUS Spohn Hospital Corpus Christi - Shoreline, Corpus Christi, USA; 4 Hospital Medicine, Sentara Northern Virginia Medical Center, Woodbridge, USA; 5 Hospital Medicine, Mary Washington Hospital, Fredericksburg, USA; 6 Medicine, Independent Researcher, Philadelphia, USA; 7 Hospital Medicine, University of Pennsylvania, Chester County Hospital, Philadelphia, USA

**Keywords:** multisystem inflammatory syndrome, complication, prevention, mis-c, side effects, covid-19 vaccine, covid-19

## Abstract

Symptoms of COVID-19 infection are usually mild in the healthy pediatric population. In some pediatric patients, COVID-19 infection can lead to multisystem inflammatory syndrome in children (MIS-C). We report two cases. Case 1 is a rare case of MIS-C symptoms, presenting with myalgia, chest pain, and fever three days after the second dose of the Pfizer-BioNTech COVID-19 vaccine, which is compared with Case 2, which is a case of MIS-C in an unvaccinated patient with COVID-19 infection who was noted to have acute kidney injury and fluid refractory hypotension. Although MIS-C was reported as a vaccine side effect, we conclude that COVID-19 infection led to the development of MIS-C in our case, not the COVID-19 vaccine. MIS-C symptoms were also noted to be less severe after the COVID-19 vaccine than in the unvaccinated patients.

## Introduction

Children infected with the coronavirus disease 2019 (COVID-19) virus usually have mild symptoms [1]. However, in some rare cases, children can be severely affected. Children with severe COVID-19 infection occasionally present with incomplete Kawasaki disease or toxic shock-like syndrome, now known as multisystem inflammatory syndrome in children (MIS-C) [2]. MIS-C affects multiple systems, and treatment for these children may require various pediatric specialists. All pediatric patients who meet MIS-C criteria should be managed in the inpatient setting. Although MIS-C is usually associated with immune dysregulation caused by the severe acute respiratory syndrome coronavirus 2 (SARS-CoV-2), children receiving COVID-19 vaccination were reported to be at risk for MIS-C because of immune-related side effects by Yousaf et al. [3].

## Case presentation

We report two cases. Case 1 patient, after her first vaccine, was exposed to COVID-19 infection from immediate family members. Case 2 was noted in a patient with COVID-19 infection, with no prior vaccination.

Case 1

A 17-year-old female presented for one day with tactile fever, nausea, myalgia, and chest pain. The patient denied cough or upper respiratory infection symptoms. The patient was exposed to COVID-19 infection from household members about two weeks prior. The patient received the second BNT162b2 (Pfizer-BioNTech) COVID-19 vaccine three days before presentation. Her first BNT162b2 vaccine was 25 days before presentation. On exam, she was febrile with no rash or no chest tenderness. On lab investigations, troponin was elevated to 3.6 ng/mL. The patient was noted to have polymerase chain reaction (PCR)-positive COVID-19. Given the recent vaccination, the test for severe acute respiratory syndrome (SARS)-COVID-19 antibodies was not done, which would have affected antibody results. C-reactive protein (CRP) was elevated to 4.5 mg/dL. D-dimer was 685 ng/ml. The EKG, echocardiogram (ECHO), thyroid-stimulating hormone, electrolytes, liver function tests, and complete blood count were within normal range.

She was started on the MIS-C treatment protocol with enoxaparin and intravenous (IV) steroids per cardiology recommendations. After treatment, troponin decreased to 0.454 ng/ml the next day. She was discharged after one day. She continued to taper steroids outpatient for a total of 15 days.

On the outpatient follow-up visit after completing the steroid and aspirin course, her cardiac exam was normal. The EKG was normal. Her echocardiogram remained normal.

Case 2

A 14-year-old African American female presented with fever, shortness of breath, lethargy, nausea, and sore throat. The patient denied pink eye, rash, or abdominal pain. The patient was not vaccinated for COVID-19. Her BMI was >99th percentile for age. Physical exam was significant for acutely ill-appearing patient with increased capillary refill time. COVID-19 PCR and antibody tests were positive. White blood cell count was elevated to 15.3 × 109/L with lymphopenia at 6%. Hemoglobin was reduced to 8.8 g/dl, along with thrombocytopenia. Troponin at admission was 0.19 ng/mL, which increased to 0.29 at its peak during follow-up labs. Chest X-ray was unremarkable. EKG showed subtle ST depression and T-wave inversion in inferior leads.

The echocardiogram showed mildly decreased left ventricular function, with ejection fraction at 54% and mild mitral and tricuspid regurgitation. Pro-B-type natriuretic peptide (pro-BNP), erythrocyte sedimentation rate (ESR), CRP, D-dimer, and fibrinogen were elevated, as shown in Table 1. The patient was started on ceftriaxone and azithromycin. The patient remained hypotensive despite adequate fluid boluses requiring initiation of norepinephrine and epinephrine drips. She was also started on MIS-C protocol with intravenous immunoglobulin (IVIG) and steroids. After completion of the treatment, repeat ECHO showed improvement of left ventricle (LV) function with ejection fraction (EF) of 63% and normal EKG. The patient was discharged from the ICU after three days.

**Table 1:** Case presentation: Case 1 and Case 2 MIS-C: multisystem inflammatory syndrome in children; RT-PCR: reverse transcription-polymerase chain reaction; CXR: chest x-ray; Pro-BNP: pro-B-type natriuretic peptide; AST: aspartate aminotransferase; ALT: alanine aminotransferase; ESR: erythrocyte sedimentation rate; CRP: C-reactive protein; IVIG: intravenous immunoglobulin; VENT: ventilation; HFNC: high-flow nasal cannula; BiPAP: bilevel positive airway pressure; EF: ejection fraction.

Clinical features	Case 1: MIS-C after COVID-19 vaccine 2nd dose	Case 2: MIS-C in unvaccinated patient
Age (years)	17	14
Sex	Female	Female
Race	Caucasian	African American
BMI percentile	92th	>99th
Comorbidities	Overweight	Obesity
Primary diagnosis	MIS-C (3 days after COVID-19 2nd vaccine)	MIS-C with acute kidney injury and fluid refractory hypotension
COVID-19 antibody	Not done	+
COVID-19 RT-PCR	+	+
COVID-19 vaccination	2 vaccine doses of Pfizer-BioNTech	None
Presenting symptoms	Myalgia, chest pain, and tactile fever	Shortness of breath, lethargy, nausea, and sore throat
Fever	+	+
Nausea/vomiting	+	+
Duration of symptoms	One day	Three days
Past/family history/allergies	None	None
Evaluation		
CXR	Normal	Normal
Electrocardiogram (EKG)	Normal	Subtle ST depression & T wave inversion in inferior lead
Troponin I (admission) (Ref: 0.0-0.08 ng/mL)	3.6	0.19
Troponin I (peak)	6	0.29
Pro-BNP (Ref: 5-363 PG/ML)	405	7361
Echocardiogram (ECHO) at admission	Normal	LV function with mildly decreased EF (54%), mild mitral and tricuspid regurgitation
Hemoglobin (Ref: 11.0-14.3 g/dl)	12.5	8.8
White cell count (Ref: 4.5-11.5 K/UL)	9	15.3
Lymphocyte % (Ref: 24-44%)	24%	6%
Platelets (×103/μL) (Ref: 150-400 × 103/μL)	325	126
Creatinine (Ref: 0-1.2 mg/dl)	0.6	1.49
AST/ALT (Ref: 0-60 U/L)	34/42	40/31
Albumin (Ref: 3.4-5 g/dl)	4.5	2.7
ESR (Ref: 0-15 mm/hr)	Not done	145
CRP (Ref: 0-0.9 mg/dl)	7.1	42
Procalcitonin (Ref: <0.16 ng/mL)	1.2	16.4
Ferritin (Ref: 13.7-78.8 ng/mL)	60	554
D-dimer (Ref: <500 ng/ml)	685	2261
Fibrinogen (Ref: 191-492 mg/dl)	357	651
Lactic acid (Ref: 0.5-2.2 mmol/l)	1.7	7.7
Treatment		
IVIG	+	+
Steroids	+	+
VENT/HFNC/BiPAP	None	None
Vasopressor	None	Norepinephrine epinephrine
Antibiotics	None	Ceftriaxone & azithromycin
Length of hospital/ICU stay	One day	Three days

Both the patient's presentation and management details are summarized and compared in Table 1.

## Discussion

MIS-C, or pediatric multisystem inflammatory syndrome (PMIS), is a condition usually seen in pediatric patients affected by COVID-19 infection [4]. Patients suspected of MIS-C show signs and symptoms of multiple organs inflamed, making this syndrome a very serious condition. The diagnostic criteria to identify MIS-C provided by the Centers for Disease Control and Prevention (CDC) have been discussed below in Table 2 [4].

**Table 2:** MIS-C diagnostic criteria Adapted from [4]. MIS-C: multisystem inflammatory syndrome in children; RT-PCR: reverse transcription-polymerase chain reaction.

MIS-C diagnostic criteria: All four of these must be met	
1. Age	<21 years
2. Clinical manifestations suggesting MIS-C	Self-reported fever > 38˚C that lasts over 24 hours.
	Laboratory findings indicating inflammation like elevated levels of C-reactive protein (CRP), erythrocyte sedimentation rate (ESR), fibrinogen, ferritin, interleukin-6 (IL-6), D-dimer, lactic acid dehydrogenase, neutrophils, and decreased lymphocyte or albumin levels.
	Hospitalization due to illness with involvement of at least two major organ systems, which might include cardiac, gastrointestinal, neurologic, pulmonary, renal, and dermatologic.
3. Exclusion of other possible diagnoses	
4. Evidence of Infection	Current COVID-19 infection positive test (via RT-PCR, antigen test, or serology) or exposure to COVID-19 four weeks before symptoms began.

There is some overlap in clinical presentation with Kawasaki disease due to patients presenting with red eyes, suggesting conjunctivitis, and swelling of the hands and feet. Patients with MIS-C who present with Kawasaki disease-like symptoms were noted to have more gastrointestinal involvement. Gastrointestinal symptoms may include nausea, vomiting, changes in bowel movements consistent with diarrhea, and abdominal pain [5]. Other symptoms may include shock, acute kidney dysfunction, and trouble breathing, suggesting respiratory or cardiovascular compromise [5]. There is wide variability in the clinical presentation of MIS-C due to the involvement of multiple organ systems. It has been reported to the VAERS (Vaccine Adverse Event Reporting System) that some patients developed MIS-C-like symptoms after receiving a COVID-19 vaccine [6].

Per Belot et al. [7], between the SARS-CoV-2 infection and MIS-C onset, a mean 28-day delay was noticed. In contrast, the first or single COVID-19 vaccine injection to MIS-C onset was reported to be a median of 25 days [8]. This study suggests that SARS-CoV-2 infection occurred before or shortly after the vaccine injection. However, Levy et al.'s [8] study has no MIS-C cases in fully vaccinated children. An analysis performed from July to December 2021 in adolescents aged 12 to 18 years estimated that two doses of the BNT162b2 vaccine (Pfizer-BioNTech) were 91% effective against MIS-C [4]. This study noted that all adolescent patients who needed life support had no prior history of COVID-19 vaccination [4]. The Incidence of MIS-C was reported in one per million individuals who received the COVID-19 vaccine, while prior investigations report MIS-C incidence of around 300 per million cases of COVID-19 infection [3,9]. In a follow-up study on BNT162b2 vaccine, two doses of BNT162b2 were correlated with a decreased likelihood of MIS-C [10].

Our case 1 developed MIS-C after two doses of the COVID-19 vaccine. Comparison with our case 2 demonstrates that MIS-C symptoms were much less severe after the vaccination. Our vaccinated patient did not develop acute kidney injury and fluid refractory hypotension and had a much shorter intensive care unit (ICU) hospital stay than the unvaccinated, suggesting the reduced risk of MIS-C after vaccination, as it has been reported in other publications [3,4].

Among patients with myocarditis, vaccine-related cases were noted to have rapid recovery of cardiac function and resolution of symptoms [11]. No serious adverse events were reported after vaccination in a multicenter cross-sectional study of patients with a history of MIS-C, those receiving COVID-19 vaccine ≥90 days after MIS-C diagnosis [12]. Given the safety profile, COVID-19 vaccination is recommended for all eligible pediatric patients [10,12,13].

Strengths and weakness

Our article reports additional cases of MIS-C in patients with COVID-19 infection or vaccination, intending to provide details and contribute to further research. The article has some weaknesses, with limitations inherent to the publication type. Our report includes two selected patient presentations with a sample selection among those hospitalized at a single health center. A non-blinded comparison between the two cases is meant to provide a contrast and cannot be used to generalize the conclusion, which will require a large sample size.

## Conclusions

Vaccination against COVID-19 was noted to reduce the severity of MIS-C in our patient, similar to other publications. Due to this condition affecting several body systems, symptoms are related to multi-organ involvement, including gastrointestinal symptoms, and the potential need for life support. These risks further emphasize the importance of pediatric vaccination and the need for further large-scale studies to understand this uncommon yet severe condition better.
